# Deacetylation of HSD17B10 by SIRT3 regulates cell growth and cell resistance under oxidative and starvation stresses

**DOI:** 10.1038/s41419-020-02763-9

**Published:** 2020-07-23

**Authors:** Lu Liu, Shuaiyi Chen, Miao Yu, Chenxu Ge, Mengmeng Ren, Boya Liu, Xin Yang, Thomas W. Christian, Ya-Ming Hou, Junhua Zou, Wei-Guo Zhu, Jianyuan Luo

**Affiliations:** 1 Department of Medical Genetics, Center for Medical Genetics, Peking University Health Science Center, Beijing, 100191 China; 2 Department of Biochemistry and Molecular Biology, Thomas Jefferson University, 233 South 10th Street, 220 BLSB, Philadelphia, PA 19107 USA; 3 Department of Biochemistry and Molecular Biology, Shenzhen University School of Medicine, Shenzhen, 518060 China; 4 Beijing Key Laboratory of Protein Posttranslational Modifications and Cell Function, Department of Biochemistry and Molecular Biology, Peking University Health Science Center, Beijing, 100191 China

**Keywords:** Cell death, Acetylation

## Abstract

17-beta-hydroxysteroid dehydrogenase 10 (HSD17B10) plays an important role in mitochondrial fatty acid metabolism and is also involved in mitochondrial tRNA maturation. HSD17B10 missense mutations cause HSD10 mitochondrial disease (HSD10MD). HSD17B10 with mutations identified from cases of HSD10MD show loss of function in dehydrogenase activity and mitochondrial tRNA maturation, resulting in mitochondrial dysfunction. It has also been implicated to play roles in the development of Alzheimer disease (AD) and tumorigenesis. Here, we found that HSD17B10 is a new substrate of NAD-dependent deacetylase Sirtuin 3 (SIRT3). HSD17B10 is acetylated at lysine residues K79, K99 and K105 by the acetyltransferase CBP, and the acetylation is reversed by SIRT3. HSD17B10 acetylation regulates its enzymatic activity and the formation of mitochondrial RNase P. Furthermore, HSD17B10 acetylation regulates the intracellular functions, affecting cell growth and cell resistance in response to stresses. Our results demonstrated that acetylation is an important regulation mechanism for HSD17B10 and may provide insight into interrupting the development of AD.

## Introduction

17-beta-hydroxysteroid dehydrogenase 10 (HSD17B10), encoded by HSD17B10, is a member of the short-chain dehydrogenase superfamily^1^. HSD17B10 is the only family member located in the mitochondria^2^. It catalyzes the beta-oxidation of fatty acids, androgens, and estrogens, contains 3-alpha-hydroxysteroid dehydrogenase activity, and carries out oxidative conversions of 7-alpha-OH and 7-beta-OH bile acids^3–5^. HSD17B10 plays an important role in mitochondrial fatty acid metabolism.

Besides mitochondrial dehydrogenase activity, HSD17B10 is also an essential subunit of human mitochondrial ribonuclease P (RNase P), a complex involved in mitochondrial tRNA maturation^6^. RNase P is a protein complex that cleaves tRNA molecules in their 5′-ends^7^. HSD17B10 (MRPP2), together with TRMT10C (MRPP1), forms a subcomplex of the mitochondrial RNase P, called the MRPP1–MRPP2 subcomplex^8^. The MRPP1–MRPP2 subcomplex acts as a tRNA maturation platform. It is involved in the 5′-end cleavage by the mitochondrial RNase P complex and is responsible for the N1-methylation of adenosine and guanosine at position 9 (m1A9 and m1G9, respectively) of human mitochondrial tRNA^8,9^. The MRPP1–MRPP2 subcomplex enhances the efficiency of mitochondrial tRNA 3′-processing and presents the nascent tRNA to the mitochondrial CCA tRNA nucleotidyltransferase to assist the maturation of mitochondrial tRNA^10^. Hence, HSD17B10 plays an important role in mitochondrial tRNA maturation.

HSD17B10, mapping at Xp11.2, is a highly conserved gene across a large evolutionary distance from nematodes to mammals, implying that HSD17B10 plays a crucial role^11^. HSD17B10 missense mutations cause HSD10 mitochondrial disease (HSD10MD), with features including progressive neurodegeneration, psychomotor retardation, loss of mental and motor skills, seizures, cardiomyopathy, and vision and hearing impairment^12^. HSD17B10 with mutations identified from cases of HSD10MD show loss of function in dehydrogenase activity and mitochondrial tRNA maturation, resulting in mitochondrial dysfunction^13–15^. HSD17B10 has a special D-loop structure that interacts with amyloid-beta (Aβ)^16^. In Alzheimer disease (AD), HSD17B10 activity is inhibited by interacting with intracellular Aβ which may contribute to the neuronal dysfunction associated with AD^17^.

Post-translational modification of proteins is of great importance in regulating protein functions. A study showed that Parkin can regulate mitochondrial abundance of HSD17B10 in a ubiquitin-dependent manner to promote mitochondrial elongation^18^. In our previous study, we identified HSD17B10 from NAD-dependent deacetylase Sirtuin 3 (SIRT3) complexes^19^. In this study, we show that HSD17B10 is deacetylated by SIRT3, and its function is regulated by its acetylation levels. Our results suggest a post-translational modification pathway that regulates the functions of HSD17B10 and results in a change of cellular phenotype.

## Materials and methods

### Protein purification

Constructs were transfected into HEK293T cells by Polyethyleneimine (PEI). After 48 h, cells were harvested and lysed in BC200 buffer (200 mM NaCl, 20 mM pH7.3 Tris, 20% glycerol, 0.2% NP-40). Cell lysates were incubated with anti-Flag M_2_ beads (Sigma, USA)/anti-HA beads (Roche, Switzerland) at 4 °C overnight. The beads were washed with BC100 four times and eluted with Flag peptide (Sigma)/HA peptide (Roche) at 4 °C for 4 h.

GST and GST fusion proteins were expressed in Rosetta (DE3) (CWBiotech, Beijing, China) bacterial cells, treated with 0.8 M IPTG (Sigma) at 37 °C for 4 h to induce fusion protein expression. Bacterial cells were harvested and suspended in 10–20 mL PBS. The same volume of BC1000 (1 M NaCl, 20 mM Tris, 40% glycerol, 2% Triton X-100) was added and bacterial cells were lysed by sonication. The lysates were incubated with GST-agarose beads (Novagen) at 4 °C overnight. The beads were washed with BC100 four times and eluted with Glutathione (GSH) at 4 °C for 4 h.

SDS-PAGE followed by Coomassie blue staining was used to quantify the amount of proteins purified from bacteria and/or cells. Bovine serum albumin (BSA) was diluted into concentration gradients, used as protein standards.

### Western blotting

HEK293T cells, HCT116 cells or U2OS cells were harvested and lysed in BC100 buffer (100 mM NaCl, 20 mM pH7.3 Tris, 20% glycerol, 0.2% NP-40). Cell lysates were incubated with anti-Flag M_2_ beads (Sigma, USA)/anti-HA beads (Roche, Switzerland) at 4 °C overnight. For endogenous immunoprecipitation, cell lysates were incubated with 1 μg anti-HSD17B10 (Abcam, UK), anti-SIRT3 (Cell Signaling Technology, USA), or normal mouse IgG, normal rabbit IgG (Santa Cruz Biotechnology, USA) at 4 °C overnight. Protein A/G agarose beads (Santa Cruz Biotechnology, USA) were added into the lysates and incubated at 4 °C for 8 h. Beads were washed with BC100 four times and eluted by 0.1 M glycine (Sigma, USA) at 4 °C for 10 min and neutralized with 1 M Tris Base. The elution was subjected to western blot and immunoblotted with indicated antibodies.

All cell lines were bought from ATCC. The information of antibodies was as follows: Anti-Flag (Sigma-Aldrich, Cat#F-3165), Anti–HA (Thermo Fisher Scientific, Cat#26183), Anti-HSD17B10 (Abcam, Cat#ab10260), Anti-SIRT3 (Cell Signaling Technology, Cat#2627 S), Anti-CBP (Cell Signaling Technology, Cat#7389 S), Anti-pan acetyllysine (Cell Signaling Technology, Cat#9441 L), Anti-β-actin (Santa Cruz Biotechnology, Cat#sc-47778), Anti-DO-1 (Cell Signaling Technology, Cat#18032 S), Anti-p62 (Proteintech, Cat#18420-1-AP), and Anti-LC3B (Sigma-Aldrich, Cat#L7543).

### GST pull-down and in vitro acetylation assay

Flag-tagged HSD17B10 and SIRT3 were purified from HEK293T cells. GST and GST fusion proteins were purified from Rosetta (DE3) bacterial cells and bound to GST-agarose beads. Flag-tagged proteins and GST fusion proteins were incubated in buffer BC200 at 4 °C overnight. Beads were washed with BC100 four times and boiled in the SDS sample buffer. The elution was subjected to western blot and immunoblotted with indicated antibodies.

For in vitro acetylation assay, the GST fusion proteins were purified from Rosetta (DE3) bacterial cells. The reaction mixture contained 10 × Ac Buffer1 (200 mM pH8.0 HEPES,10 mM DTT, 10 mM PMSF, 1 mg/ml BSA), acetyl-CoA (17 ng/μl), CBP and GST fusion proteins. The reaction mixture was incubated at 37 °C for 2 h and boiled in the SDS sample buffer.

### In vitro deacetylation assay

Flag-tagged HSD17B10 and SIRT3 were purified from HEK293T cells. The reaction mixture contained 10×Ac Buffer1, 2 × DeAc Buffer2 (8 mM MgCl_2_, 100 mM NaCl, 20% glycerol), and acetylated HSD17B10. SIRT3 and NAD^+^ (1 mM) were present or absent as needed. The reaction mixture was incubated at 37 °C for 2 h and boiled in the SDS sample buffer.

### HSD17B10 enzymatic activity assays

HSD17B10 dehydrogenase activity is measured in the reverse direction for the reduction of acetoacetyl-CoA to L-3-hydroxyacyl-CoA accompanied by the oxidation of NADH to NAD^+ ^^20^. The assay mixture contains 0.1 M pH7.0 potassium phosphate, 0.2 mg/ml bovine serum albumin, 0.2 mM NADH, and various concentrations of acetoacetyl-CoA (0–300 μM). The assay was started by the addition of 50 nM HSD17B10. The decrease in absorbance at 340 nm was measured. NADH standard curve was established to measure substrate conversion. Kinetic parameters were calculated from initial-velocity by direct curve fitting using non-linear regression analysis, and Michaelis–Menten plots were generated via GraphPad Prism 7.0.

### Generation of stable HSD17B10/SIRT3 CRISPR-Cas9 cell lines

Single guide RNA (sgRNA) sequences were as follows:

SIRT3: 5′-CACCGCTCTACACGCAGAACATCGA-3′;

HSD17B10 1#: 5′-CACCGCCACGGCGGAGCGA CTTGT-3′;

HSD17B10 5#: 5′-CACCGCATGCCCACTATTCCCCCCT-3′

sgRNAs were cloned into LentiCRISPR-V2 vector, and the plasmids were transfected with packaging plasmids pSPAX2 and pMD.2 G (at 4:3:1 ratio) into HEK293T cells. The medium was changed after 8–10 h. Viruses were collected at 48 h post-transfection to infect target cells (U_2_OS or HCT116). The infected cells were selected for stable cell lines with 1 μg/ml puromycin for 2 weeks.

### Knockdown of CBP

Small interfering RNA (siRNA) sequences were as follows:

1#: CGGCACAGCCUCUCAGUCA(dTdT)

2#: GAGGUCGCGUUUACAUAAA(dTdT)

siRNAs were transfected into HEK293T cells by RNAiMAX (ThermoFisher, USA). The medium was changed after 24 h. Cells were harvested at 48 h post-transfection and lysed in BC100 buffer (100 mM NaCl, 20 mM pH7.3 Tris, 20% glycerol, 0.2% NP-40). Cell lysates were incubated with anti-acetyllysine agarose (Immunechem, Canada) at 4 °C overnight. Beads were washed with BC100 four times and eluted by 0.1 M glycine (Sigma, USA) at 4 °C for 10 min and neutralized with 1 M Tris Base. The elution was subjected to western blot and immunoblotted with indicated antibodies.

### Measurement of cellular NAD^+^/NADH levels

NAD^+^/NADH levels in HCT116 cells were measured with the NAD/NADH Quantification Kit (Sigma, USA). 2 × 10^5^ cells were collected for each assay. Cells were extracted and samples were deproteinized before use in assay. Samples were divided into two parts for NAD_total_ and NADH only measurement. The absorbance at 450 nm (*A*_450_) was measured. All the samples were run in triplicate. NAD^+^/NADH = (NAD_total_ − NADH)/NADH.

### RT-qPCR

Total RNA was extracted using Trizol reagent (Sigma, USA). 2 μg RNA was reverse-transcribed into cDNA using the Revertra ace qPCR RT Kit (TOYOBO, Japan). Real-time PCR was performed in triplicate using AceQ qPCR SYBR green master mix (Vazyme, Nanjing, China) by the ABI 7500/7500 fast Real-Time PCR systems (Applied Biosystems, ThermoFisher, USA). The amount of tRNA was calculated by △△Ct method. The human 18S RNA was used as the internal control. For pre-tRNAs, primers were designed to span the cleavage sites. For mature tRNAs, primers were designed within gene exon, and therefore amplified both pre- and mature mitochondrial tRNAs. The primers were as follows:

mt pre-tRNA^Pro^: 5′-GATGGGTGAGTCAATACTTGGG-3′ and 5′-CCATTAGCACCCAAAGCTAAGA-3′; mt pre-tRNA^Val^: 5′-AGAGGAGACAAGTCGTAACATGG-3′ and 5′-GGTGCTTTGTGTTAAGCTACACTCTGG-3′; mt pre-tRNA^Ile^: 5′-GCGAATTCAATCTCCAGCA-3′ and 5′-GAAATAAGGGGGTTTAAGC-3′; mt tRNA^Pro^: 5′-TCAGAGAAAAAGTCTTTAACTCCACC-3′ and 5′-ATAGTTTAAATTAGAATCTTAGCTTTGGGT-3′; mt tRNA^Val^: 5′-GTGTAGCTTAACACAAAGCACCC-3′ and 5′-TCAGAGCGGTCAAGTTAAGTTG-3′; mt tRNA^Ile^: 5′-TCTTTATACAGACTATTTTC-3′ and 5′-TAGAAATAAGGGGGTTTAAG-3′; 18S RNA: 5′-GCCGCTAGAGGTGAAATTCTT-3′ and 5′-CTTTCGCTCTGGTCCGTCTT-3′.

### Mitochondrial membrane potential

HCT116 cells were grown in glass bottom cell culture dish (φ20 mm, Nest, China) to 70% confluent. 200 nM Mito Tracker Green (Invitrogen, USA) and 10 nM TMRM (ThermoFisher, USA) were incubated with cells for 30 min, followed by washing twice with HBSS media. Cells were imaged live by confocal microscopy and the intensity of fluorescence (ex: 490 nm, em: 516 nm for Mito Tracker Green; ex: 490 nm, em: 550 nm for TMRM) was recorded to determine the uncollapsed proton gradient to measure mitochondrial membrane potential.

### Cell growth

HCT116 cells were seeded into six-well plates and were counted every 24 h to establish cell growth curves. HCT116 cells were seeded into 6 cm dishes. After culturing for 7 days, cells were washed with PBS three times, fixed with 4% paraformaldehyde, and stained with 0.2% crystal violet. Pictures were shot to show colony formation. HCT116 cells were seeded into 96-well plates and cultured for 48 h. 10 μl CCK-8 was added into 100 μl DMEM (no phenol red, Gibco, USA) in each well. The plate was incubated for 1 h in the incubator (37 °C, 5% CO_2_). The absorbance at 450 nm (*A*_450_) was finally measured using microplate reader.

### Statistical analysis

**Fig. 1:**
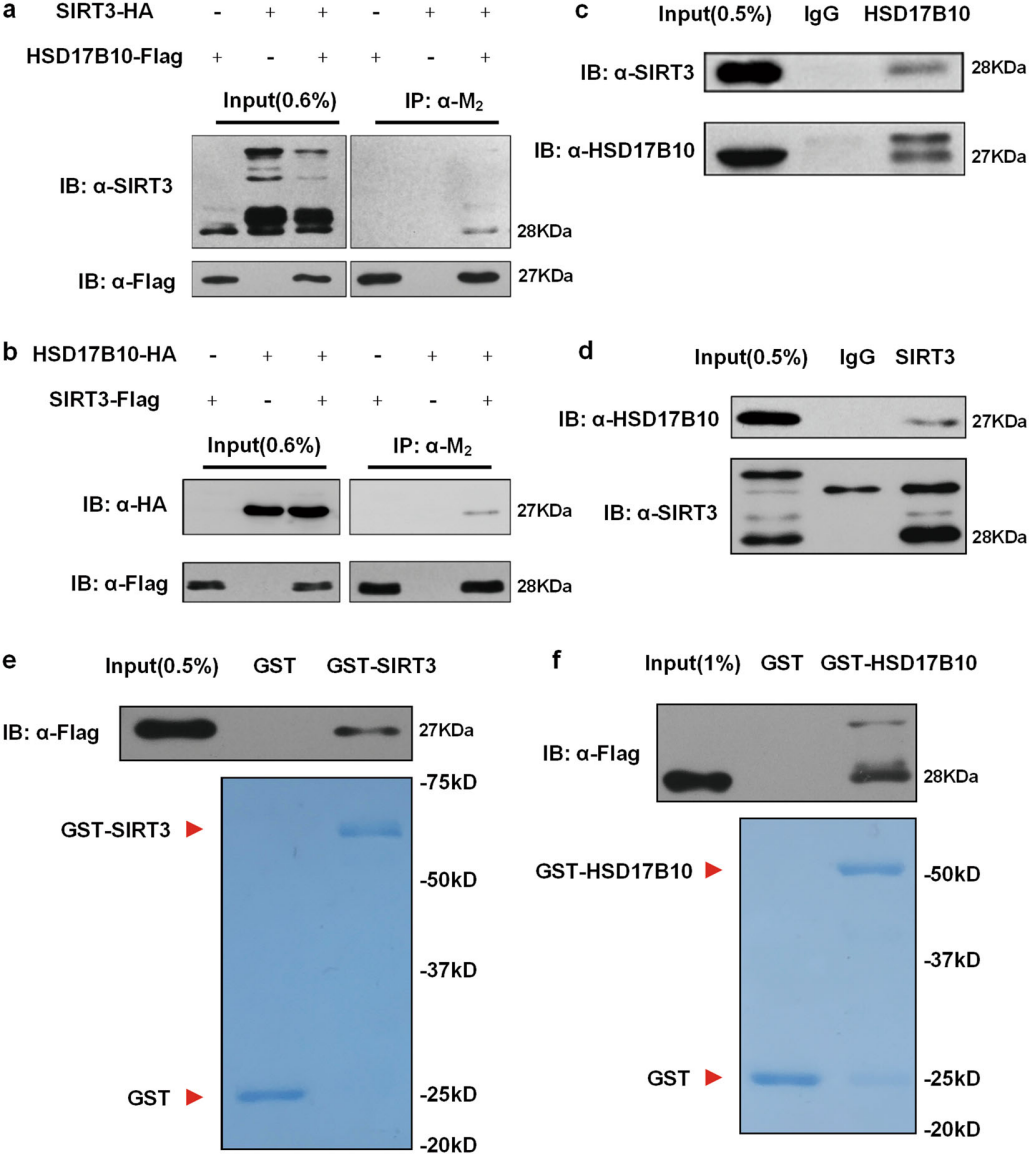
HSD17B10 interacts with SIRT3. **a** Flag-tagged HSD17B10 and HA-tagged SIRT3 were transfected individually or together into HEK293T cells followed by immunoprecipitation with M_2_ beads. **b** Flag-tagged SIRT3 and HA-tagged HSD17B10 were transfected individually or together into HEK293T cells followed by immunoprecipitation with M_2_ beads. **c** Endogenous SIRT3 was IPed from HCT116 cells. Rabbit IgG was used as a control. **d** Endogenous HSD17B10 was IPed from HCT116 cells. Mouse IgG was used as a control. **e** Flag-tagged HSD17B10 was transfected into HEK293T cells followed by immunoprecipitation with M_2_ beads. Purified Flag-tagged HSD17B10 was incubated with GST/GST-SIRT3 and was IPed with GST-agarose beads. **f** Flag-tagged SIRT3 was transfected into HEK293T cells followed by immunoprecipitation with M_2_ beads. Purified Flag-tagged SIRT3 was incubated with GST/GST-HSD17B10 and was IPed with GST-agarose beads.

**Fig. 2:**
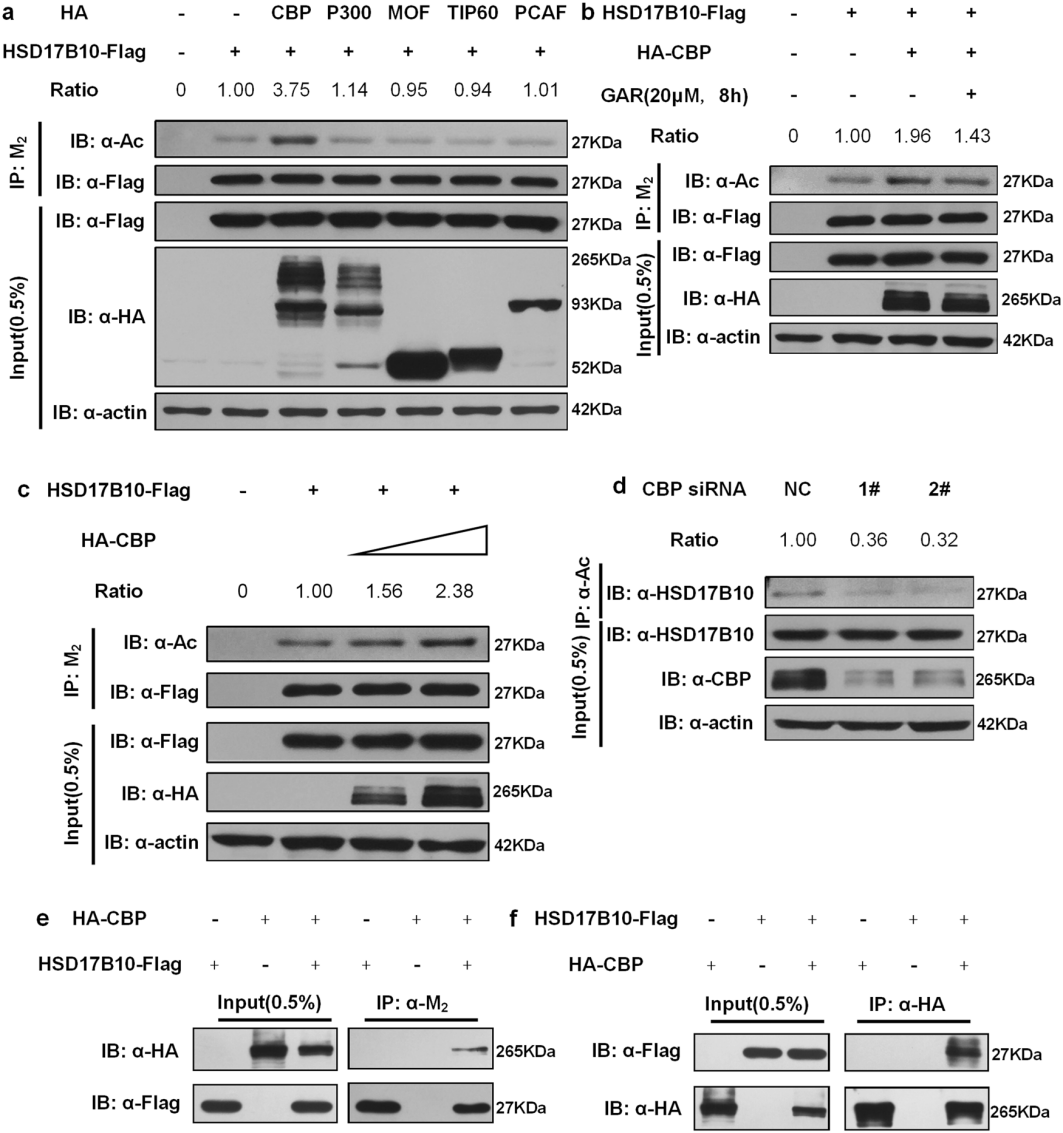
HSD17B10 can be acetylated by CBP. **a** Flag-tagged HSD17B10 was co-transfected with vector or HA-tagged acetylase CBP, P300, MOF, TIP60, or PCAF respectively as indicated. Relative HSD17B10 acetylation ratios were calculated after normalizing against Flag. **b** Flag-tagged HSD17B10 was co-transfected with vector or HA-tagged CBP. Cells were treated with GAR (20 μM) for the indicated time. Relative HSD17B10 acetylation ratios were calculated after normalizing against Flag. **c** Flag-tagged HSD17B10 was co-transfected with different amounts of HA-tagged CBP as indicated. Relative HSD17B10 acetylation ratios were calculated after normalizing against Flag. **d** CBP siRNA sequences were transfected into HEK293T cells. Endogenous HSD17B10 was purified with anti-acetyllysine agarose, and acetylated HSD17B10 levels were determined by western blot analysis. **e** Flag-tagged HSD17B10 and HA-tagged CBP were transfected individually or together into HEK293T cells followed by immunoprecipitation with M_2_ beads. **f** HA-tagged CBP and Flag-tagged HSD17B10 were transfected individually or together into HEK293T cells followed by immunoprecipitation with HA beads. The quantifications in **a**–**d** refer to the presented blots.

Experiments in Figs. 1 and 2e, f were performed twice. The other experiments were performed three times. The repeated experiments showed similar results. Sample sizes were regularly chosen as three replicates. Statistical analysis was performed via Graphpad prism 7.0. Data were expressed as mean ± standard deviation (S.D.) and S.D. was calculated to estimate the variation within each group of data.

## Results

### HSD17B10 interacts with SIRT3

In our previous report, we purified SIRT3 complex by immunoprecipitation and mass spectrometry. HSD17B10 was found in the complex^19^. To verify the interaction between HSD17B10 and SIRT3, we overexpressed tagged SIRT3 and HSD17B10 in HEK293T cells and performed co-immunoprecipitation assays. SIRT3 can interact with HSD17B10 (Fig. 1a), and vice versa (Fig. 1b). SIRT3-HSD17B10 interaction was further confirmed by the co-immunoprecipitation of endogenous SIRT3 and HSD17B10 (Fig. 1c, d). We also performed GST pull-down assay to determine if HSD17B10 and SIRT3 can directly interact with each other. The results showed that GST-SIRT3 pulled down HSD17B10 (Fig. 1e) and GST-HSD17B10 pulled down SIRT3 (Fig. 1f). Taken together, we demonstrated that HSD17B10 directly interacts with SIRT3 both in vivo and in vitro.

### HSD17B10 is acetylated by CBP

The interaction between HSD17B10 and SIRT3 prompted us to consider whether HSD17B10 is a novel target of SIRT3. Therefore, we first examined whether HSD17B10 could be acetylated. We co-transfected Flag-HSD17B10 with different acetyltransferases in HEK293T cells and incubated the cell lysates with M_2_ beads to immunoprecipitate HSD17B10. HSD17B10 acetylation was detected when co-transfected with CBP or p300, and CBP gave a stronger acetylation signal (Fig. 2a). Hence, CBP is the major acetyltransferase for HSD17B10. Garcinol (GAR) is a common CBP acetylation activity inhibitor. Treating cells with GAR for the indicated time inhibited HSD17B10 acetylation by CBP (Fig. 2b). Flag-tagged HSD17B10 co-transfected with different amounts of HA-tagged CBP showed that HSD17B10 acetylation increased as the amount of CBP increased (Fig. 2c). siRNA knockdown of CBP in HEK293T cells decreased acetylation levels of HSD17B10 (Fig. 2d). Flag-tagged HSD17B10 and HA-tagged CBP transfected individually or together into HEK293T cells followed by immunoprecipitation showed that HSD17B10 and CBP could interact with each other (Fig. 2e, f). In conclusion, we demonstrated that HSD17B10 can be acetylated and CBP is the major acetyltransferase.

### HSD17B10 is deacetylated by SIRT3

**Fig. 3:**
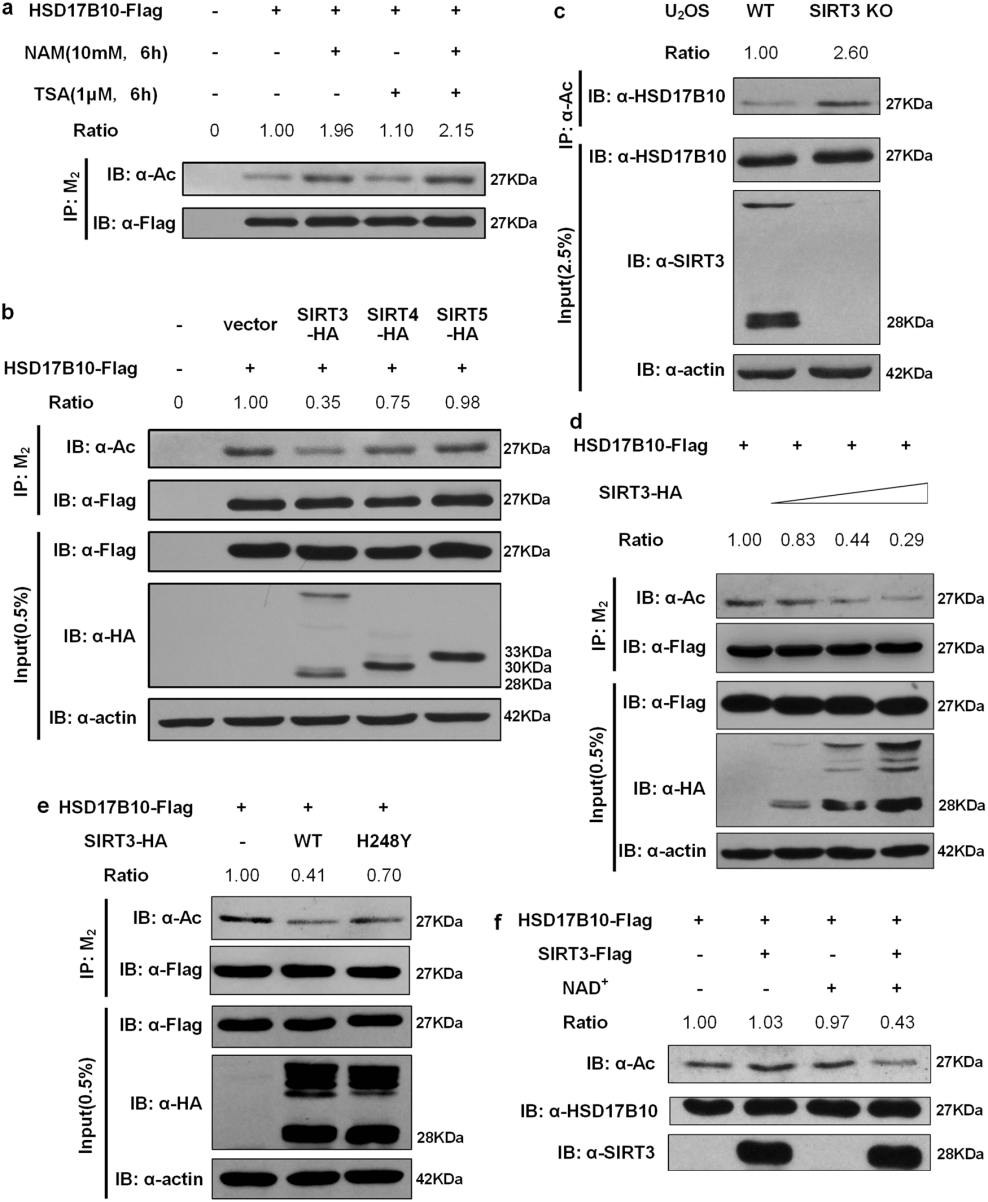
SIRT3 is the main deacetylase for HSD17B10. **a** Flag-tagged HSD17B10 was transfected into HEK293T cells, and cells were treated with 10 mM NAM and/or 1 μM TSA for the indicated time. Relative HSD17B10 acetylation ratios were calculated after normalizing against Flag. **b** Flag-tagged HSD17B10 was co-transfected with vector or HA-tagged SIRT3/SIRT4/SIRT5 respectively as indicated. Relative HSD17B10 acetylation ratios were calculated after normalizing against Flag. **c** Endogenous HSD17B10 was purified with anti-acetyllysine agarose in WT or SIRT3 knockout U2OS cells. Acetylated HSD17B10 levels were determined by western blot analysis. **d** Flag-tagged HSD17B10 was co-transfected with different amounts of HA-tagged SIRT3 as indicated. Relative HSD17B10 acetylation ratios were calculated after normalizing against Flag. **e** Flag-tagged HSD17B10 was co-transfected with vector, HA-tagged WT SIRT3 or the catalytic center mutation SIRT3 H248Y respectively. Relative HSD17B10 acetylation ratios were calculated after normalizing against Flag. **f** Flag-tagged HSD17B10 and SIRT3 were respectively transfected into HEK293T cells and were purified with M_2_ beads. In vitro deacetylation assay was performed as indicated. Relative HSD17B10 acetylation ratios were calculated after normalizing against Flag. The quantifications in this figure refer to the presented blots.

We further explored if SIRT3 can deacetylate HSD17B10. We transfected Flag-tagged HSD17B10 into HEK293T cells and treated cells with nicotinamide (NAM) and Trichostatin A (TSA) individually or simultaneously for the indicated time. We found that HSD17B10 acetylation levels were higher after treating cells with NAM, a specific inhibitor of the sirtuins, whereas treating with TSA, an inhibitor of other HDACs, did not affect HSD17B10 acetylation (Fig. 3a). Meanwhile, treating with NAM together with TSA did not further enhance HSD17B10 acetylation compared to treating with NAM alone (Fig. 3a). These results indicate that HSD17B10 acetylation is regulated by the sirtuins family.

Study on HSD17B10 subcellular location shows that it is distributed in mitochondria^2^. SIRT3, SIRT4, and SIRT5 are three mitochondrial members of the sirtuins family. Thus, we co-transfected Flag-tagged HSD17B10 together with HA-tagged SIRT3, SIRT4, or SIRT5 into HEK293T cells. Compared to SIRT4 or SIRT5, SIRT3 gave the strongest deacetylation activity of HSD17B10 (Fig. 3b). Knockout of SIRT3 in U2OS cells showed increased acetylation levels of HSD17B10 (Fig. 3c), indicating that HSD17B10 is hyperacetylated in SIRT3 knockout cells. Flag-tagged HSD17B10 co-transfected with different amounts of HA-tagged SIRT3 showed that HSD17B10 acetylation decreased as the amount of SIRT3 increased (Fig. 3d). We found that SIRT3 H248Y, a SIRT3 catalytic center mutant, cannot deacetylate HSD17B10 as much as SIRT3 WT (Fig. 3e). In addition, we conducted an in vitro deacetylation assay and found that SIRT3 can deacetylate HSD17B10 in vitro and the deacetylation process is NAD^+^-dependent (Fig. 3f). Thus, we concluded that SIRT3 can deacetylate HSD17B10 both in vivo and in vitro.

### Identify the major acetylation sites of HSD17B10

**Fig. 4:**
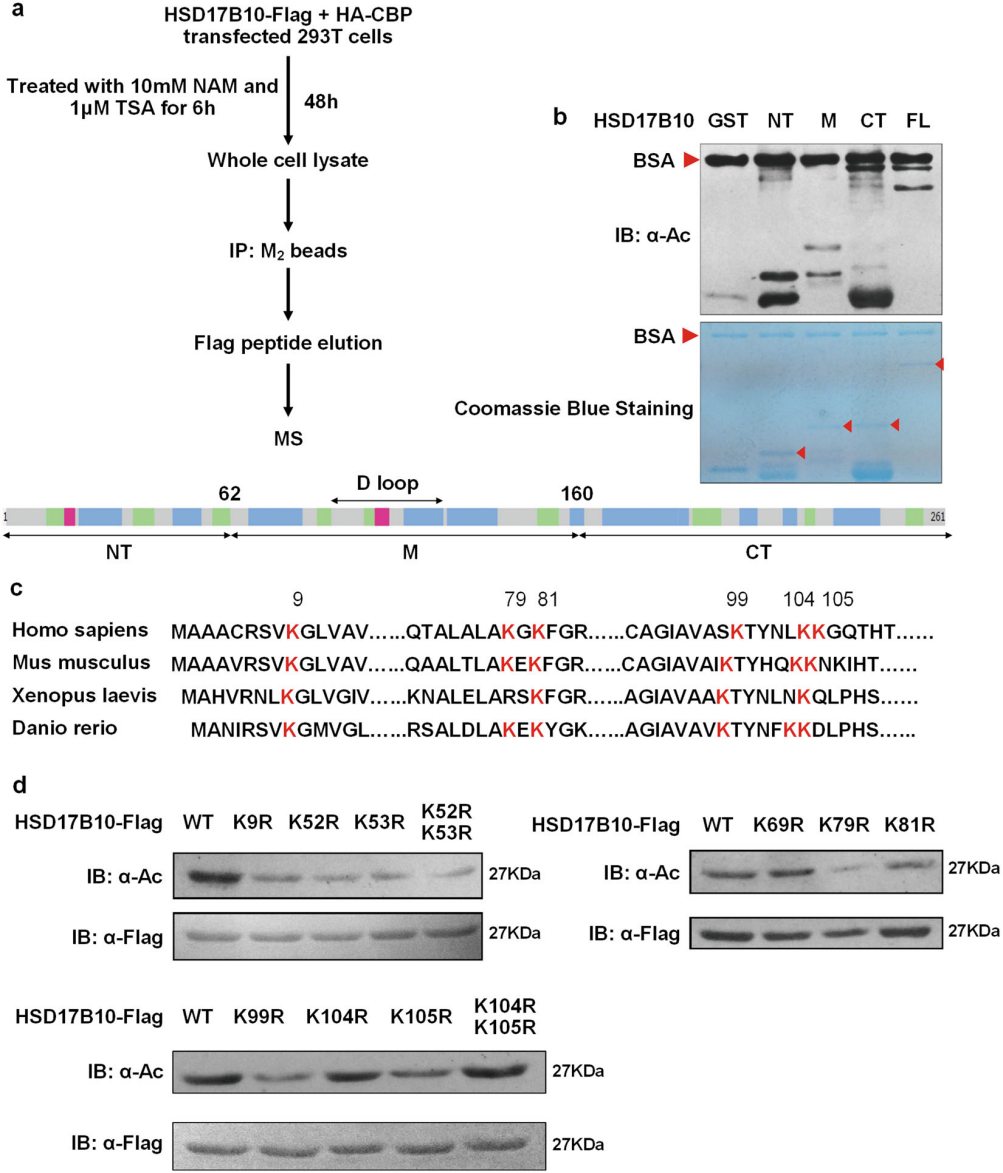
Identify the main acetylation sites of HSD17B10. **a** Flag-tagged HSD17B10 was co-transfected with HA-tagged CBP, and cells were treated with 10 mM NAM for 6 h. Whole-cell lysate was collected, IPed with M_2_ beads and eluted by Flag peptide. After SDS-PAGE gel separating and Coomassie blue staining, the specific band for HSD17B10 was analyzed by mass spectrometry. **b** HSD17B10 GST N-terminal (NT), middle (M), C-terminus (CT) and full-length (FL) plasmids were constructed, and in vitro acetylation assay was conducted. GST protein was used as a negative control. **c** HSD17B10 amino acid sequence conservation was analyzed via sequence analyzing software MEGA. **d** HSD17B10 KR mutant (mimicking the deacetylation status) plasmids were constructed. Flag-tagged HSD17B10 WT and KR mutants were individually transfected and cells were treated with 10 mM NAM for 6 h. Relative HSD17B10 acetylation ratios were calculated after normalizing against Flag.

To identify the major acetylation sites of HSD17B10, we purified acetylated HSD17B10 for mass spectrometry (MS) (Fig. 4a). The result showed that K52, K53, K99, K105 and K172 are putative acetylation sites. We further conducted an in vitro acetylation assay. HSD17B10 was purposely separated into three fractions as indicated in Fig. 4b. HSD17B10 GST fusion protein N-terminus (NT), middle (M) and full-length (FL) showed acetylation signal but not C-terminus (CT), indicating that acetylation sites of HSD17B10 are at NT and M fractions, which also excluded CT located lysine residues K172 and K212. HSD17B10 is a highly evolutionary conservation protein^11^, and conserved lysine residue usually plays a critical role in protein acetylation and can affect protein functions. We analyzed HSD17B10 amino acid sequence conservation and identified K9, K79, K81, K99, K104 and K105 as highly conserved lysines (Fig. 4c). We constructed KR mutant plasmid which is a mimic for deacetylation status, and transfected WT and KR mutant HSD17B10 into HEK293T cells to examine their acetylation status. We found that K9R, K52R, K53R, K79R, K99R and K105R decreased HSD17B10 acetylation levels (Fig. 4d). Altogether, these data showed that K9, K52, K53, K79, K99 and K105 are putative acetylation lysine residues of HSD17B10.

### HSD17B10 acetylation affects its enzymatic activity and the formation of mt-tRNA splicing complex

**Fig. 5:**
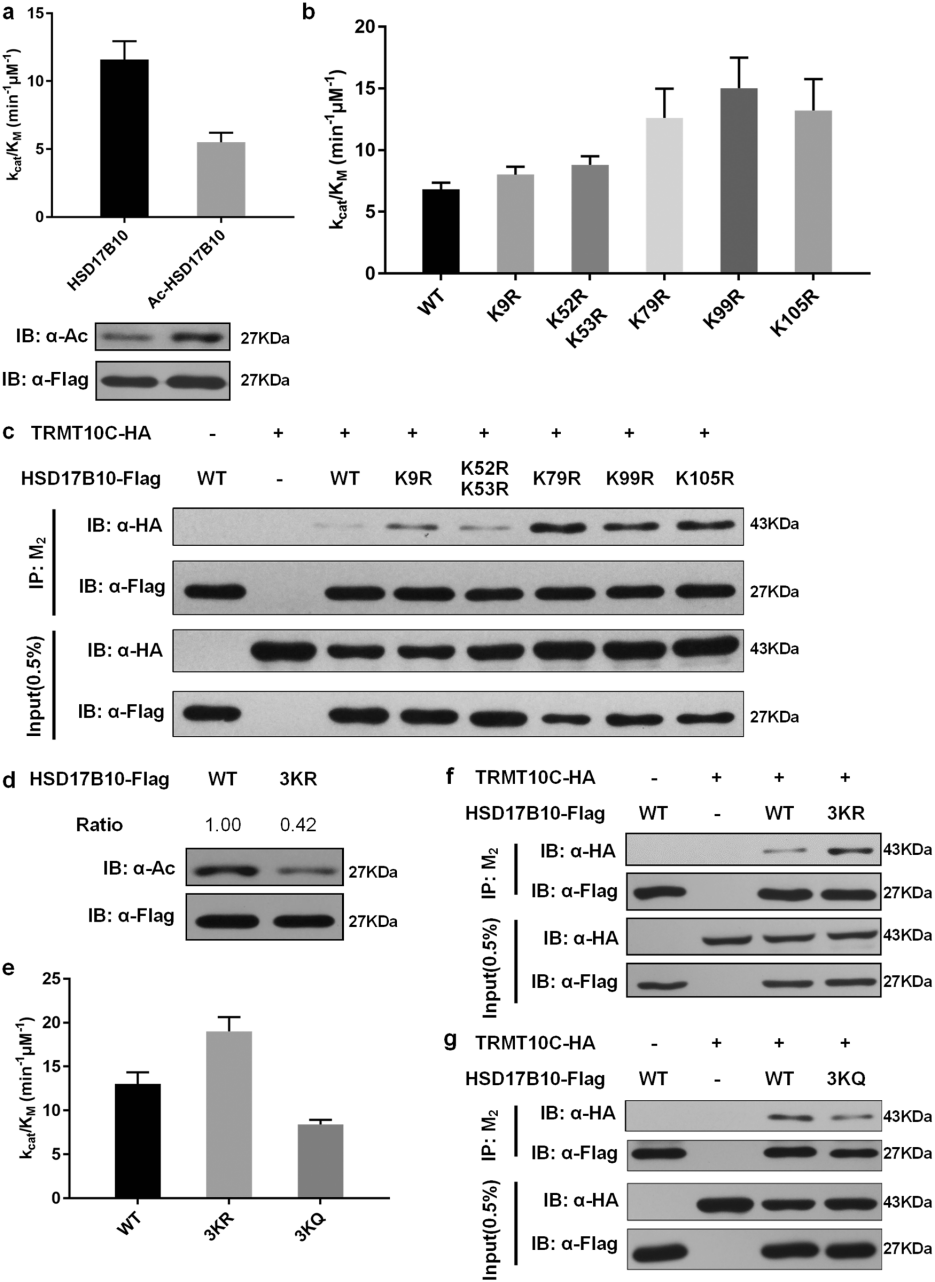
HSD17B10 acetylation affects its enzymatic activity and the formation of mt-tRNA splicing complex. **a** Flag-tagged HSD17B10 was transfected into HEK293T cells, and cells were treated with or without 10 mM NAM for 6 h for deacetylated and hyperacetylated HSD17B10 purification. The L-3-hydroxyacyl-CoA dehydrogenase activity assay of HSD17B10 was conducted with acetoacetyl-CoA as the substrate and NADH as the cofactor, and changes of the absorbance at 340 nm were measured to show the decrease of NADH. *k*_cat_/*K*_M_ was calculated to represent catalytic efficiency. Data were shown as mean ± S.D. (*n* = 3). **b** Flag-tagged HSD17B10 WT and KR mutants were individually transfected, and cells were treated with 10 mM NAM for 6 h. The L-3-hydroxyacyl-CoA dehydrogenase activity of HSD17B10 was measured as previously described. Data were shown as mean ± S.D. (*n* = 3). **c** Flag-tagged HSD17B10 WT and KR mutants were co-transfected with HA-tagged TRMT10C into HEK293T cells followed by immunoprecipitation with M_2_ beads. **d** Flag-tagged HSD17B10 WT and 3KR were transfected into HEK293T cells, and cells were treated with 10 mM NAM for 6 h. Relative HSD17B10 acetylation ratios were calculated after normalizing against Flag. **e** Flag-tagged HSD17B10 WT, 3KR and 3KQ were individually transfected. The L-3-hydroxyacyl-CoA dehydrogenase activity of HSD17B10 was measured as previously described. Data were shown as mean ± S.D. (*n* = 3). **f** Flag-tagged HSD17B10 WT and 3KR were co-transfected with HA-tagged TRMT10C into HEK293T cells followed by immunoprecipitation with M_2_ beads. **g** Flag-tagged HSD17B10 WT and 3KQ were co-transfected with HA-tagged TRMT10C into HEK293T cells followed by immunoprecipitation with M_2_ beads.

HSD17B10 contains mitochondrial dehydrogenase activity^1^, and acts as a component of mt-tRNA splicing complex mitochondrial ribonuclease P^6^. We investigated if HSD17B10 acetylation regulates these functions. We purified deacetylated and hyperacetylated HSD17B10 to carry out an in vitro enzymatic activity assay^20^. Kinetic parameters were calculated from initial-velocity by direct curve fitting using non-linear regression analysis. Michaelis–Menten plots and kinetic parameters were shown in Fig. S1. *k*_cat_/*K*_M_ was calculated to represent catalytic efficiency. Acetylation of HSD17B10 significantly reduced its enzymatic activity compared to deacetylated HSD17B10 (Fig. 5a). We then purified HSD17B10 WT and KR mutants which are putative acetylated lysine residues of HSD17B10 (Fig. 4d) for the in vitro enzymatic activity assay. Interestingly, we found that only K79R, K99R and K105R dramatically increased HSD17B10 enzymatic activity (Fig. 5b). Meanwhile, in terms of interaction with TRMT10C, a component of mitochondrial ribonuclease P, HSD17B10 WT and KR mutants showed similar results. K79R, K99R and K105R mutants promoted interaction between TRMT10C and HSD17B10 more strikingly (Fig. 5c).

Thus, we concluded that K79R, K99R and K105R are three major functional acetylation lysine residues and constructed multiple lysine mutant plasmids 3KR and 3KQ (mimic for hyperacetylation status) for further investigation. We transfected WT and 3KR mutant HSD17B10 into HEK293T cells treated with SIRT3 inhibitor NAM, and 3KR acetylation levels were much reduced (Fig. 5d). We then purified WT, 3KR and 3KQ HSD17B10 for in vitro enzymatic activity assay, and HSD17B10 3KR mutant significantly enhanced its enzymatic activity compared to WT while 3KQ had the opposite effect (Fig. 5e). Co-IP assay showed the same result that 3KR mutant promoted interaction between TRMT10C and HSD17B10 whereas 3KQ acted inversely (Fig. 5f, g). Taken together, the above data demonstrated that K79, K99 and K105 are three major functional acetylation lysine residues of HSD17B10, and HSD17B10 acetylation affects its enzymatic activity and the formation of mt-tRNA splicing complex.

### HSD17B10 acetylation regulates the intracellular functions

To further investigate the physiological functions of HSD17B10 acetylation, we first established HSD17B10 CRISPR-Cas9 knockout HCT116 cells. Knocking out HSD17B10 in mice is embryonic lethal^21^, and we found that knockout of HSD17B10 in cells also caused cell death. Therefore, we only obtained HSD17B10 knockdown clones (Fig. S2a). HSD17B10 functions as a mitochondrial dehydrogenase that catalyzes the beta-oxidation of androgens, estrogens and fatty acids^3–5^. As a result, NAD^+^ will be changed to NADH. Consistent with this, our results showed that the NAD^+^/NADH ratio increased in HSD17B10 knockdown cells (Fig. S2b). Since knocking down HSD17B10 impaired the formation of mt-tRNA splicing complex, we chose mt pre-tRNA^Pro^, mt pre-tRNA^Val^, and mt pre-tRNA^Ile^ as three representative substrates. The amounts of these pre-tRNAs increased, as expected (Fig.S2c). Cells of HSD17B10-deficient patients exhibit the loss of function in dehydrogenase activity and mitochondrial tRNA maturation, resulting in mitochondrial dysfunction^13–15^. Additionally, HSD17B10 has been proved to play a protective role for mitochondrial function^18,21,22^. Therefore, we used tetramethylrhodamine methyl ester (TMRM) staining intensity as an indicator for mitochondrial membrane potential. TMRM is a cell-permeant, cationic, red-orange fluorescent dye that is readily sequestered by active mitochondria, and in dysfunctional mitochondria TMRM is released into cytoplasm and the signal will disappear. We observed that, in HSD17B10 knockdown cells, TMRM staining intensity decreased, indicating that mitochondrial function was impaired (Fig. S2d).

**Fig. 6:**
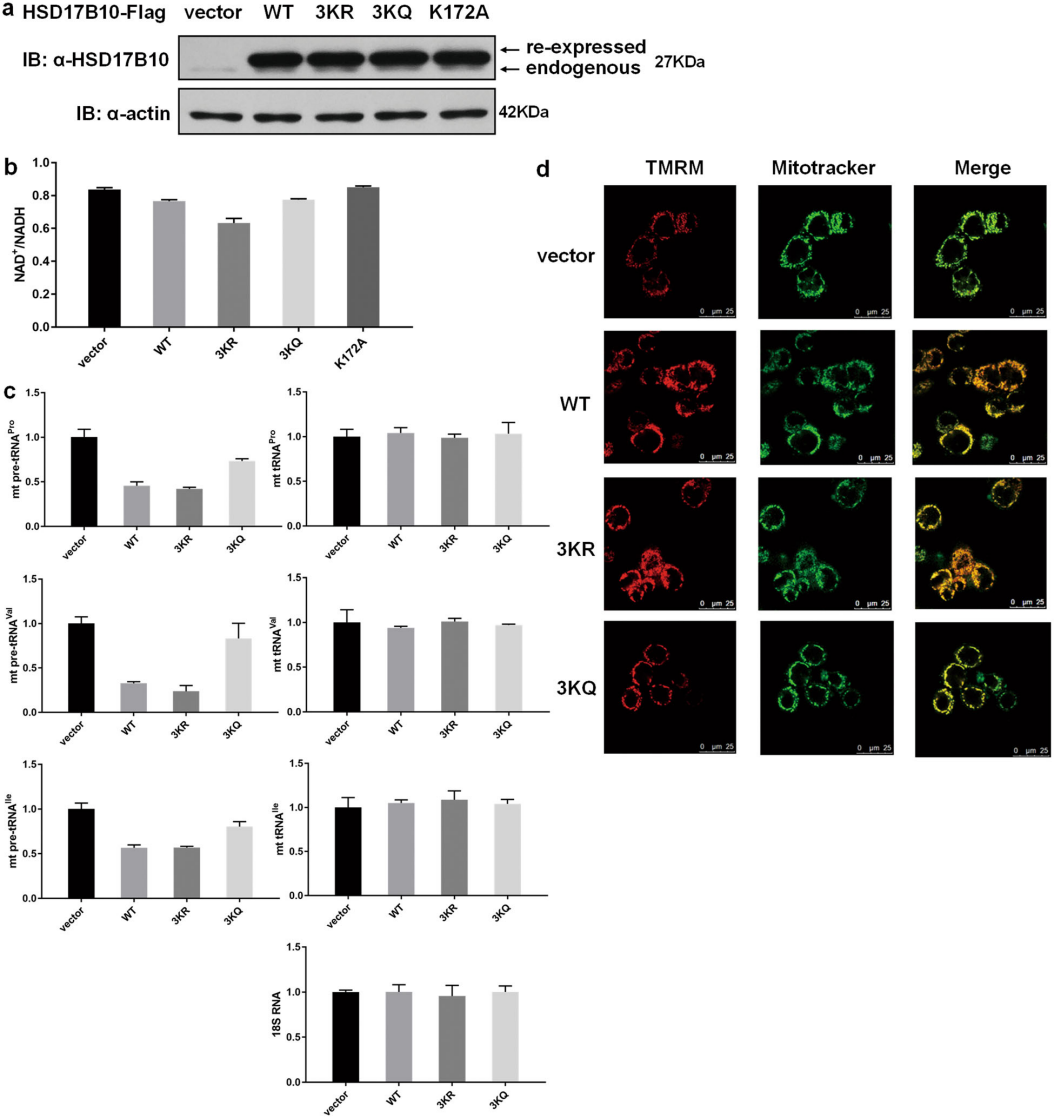
HSD17B10 acetylation regulates the intracellular functions. **a** Vector, Flag-tagged HSD17B10 WT, 3KR, 3KQ and K172A were individually transfected into HSD17B10 knockdown HCT116 cells. Endogenous and re-expressed HSD17B10 were detected with HSD17B10 antibody. **b** The absorbance at 450 nm was measure to quantify NADH and NAD_total_ in HSD17B10 knockdown HCT116 re-expressed with vector, Flag-tagged HSD17B10 WT, 3KR and 3KQ cells. NAD^+^/NADH = (NAD_total_ − NADH)/NADH. Data were shown as mean ± S.D. (*n* = 3). K172A was used as an enzymatic negative control. **c** The amounts of representative mt pre- and total tRNAs in HSD17B10 knockdown HCT116 re-expressed with vector, Flag-tagged HSD17B10 WT, 3KR and 3KQ cells were confirmed by qPCR. Data were shown as mean ± S.D. (*n* = 3). **d** HSD17B10 knockdown HCT116 re-expressed with vector, Flag-tagged HSD17B10 WT, 3KR, and 3KQ cells were stained with TMRM (10 nM) and Mitotracker (200 nM) for 0.5 h.

We then re-expressed empty vector, HSD17B10 WT, 3KR and 3KQ in HSD17B10 knockdown HCT116 cells (Fig. 6a) to investigate whether HSD17B10 acetylation affected its intracellular functions. To determine the effect of HSD17B10 enzymatic activity on cellular NAD^+^/NADH ratio, HSD17B10 K172A was served as an enzymatic negative control^8^. In Fig. 5, we demonstrated that HSD17B10 acetylation affects its enzymatic activity and the formation of mt-tRNA splicing complex. As expected, re-expressing WT HSD17B10 decreased NAD^+^/NADH ratio compared to vector, and 3KR mutant further reduced the ratio, while 3KQ barely changed the ratio compared to WT (Fig. 6b). Also, as shown in Fig. 6c, the amounts of mt pre-tRNA^Pro^, mt pre-tRNA^Val^ and mt pre-tRNA^Ile^ decreased with the re-expression of HSD17B10 WT and 3KR, while 3KQ only slightly changed their amounts compared to empty vector. Consistently, mitochondrial function was rescued by HSD17B10 WT and 3KR, while 3KQ had less effect (Fig. 6d). Taken together, we demonstrated that HSD17B10 acetylation regulated the mitochondrial function.

### HSD17B10 acetylation affects cell growth and cell resistance under stresses

**Fig. 7:**
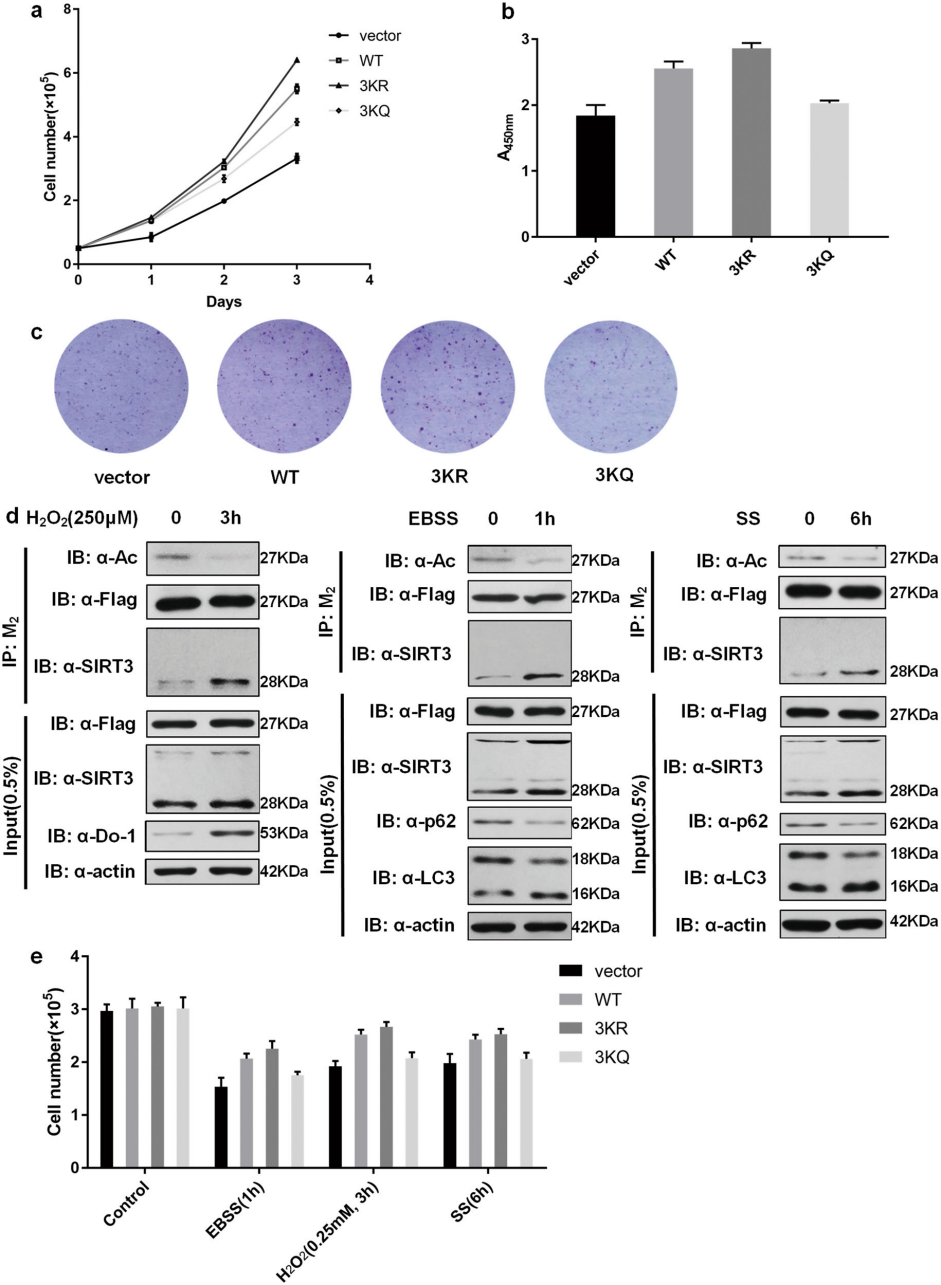
HSD17B10 acetylation affects cell growth and cell resistance under stresses. **a** 5 × 10^4^ HSD17B10 knockdown HCT116 re-expressed with vector, Flag-tagged HSD17B10 WT, 3KR and 3KQ cells were seeded into six-well plates. Cell number was counted every 24 h. Data were shown as mean ± S.D. (*n* = 3). **b** 5 × 10^3^ HSD17B10 knockdown HCT116 re-expressed with vector, Flag-tagged HSD17B10 WT, 3KR and 3KQ cells were seeded into 96-well plates. Cell growth was measured by CCK-8 assay after 48 h. Data were shown as mean ± S.D. (*n* = 3). **c** 5 × 10^3^ HSD17B10 knockdown HCT116 re-expressed with vector, Flag-tagged HSD17B10 WT, 3KR and 3KQ cells were seeded into 6 cm plates. Colony formation was shown after crystal violet staining. **d** Flag-tagged HSD17B10 was transfected into HCT116 cells, and cells were treated with different stresses for the indicated time. HSD17B10 was IPed with M_2_ beads. **e** 2 × 10^5^ HSD17B10 knockdown HCT116 re-expressed with vector, Flag-tagged HSD17B10 WT, 3KR and 3KQ cells were seeded into six-well plates. After 24 h, cells were treated with different stresses for the indicated time, and the number of living cells was counted. Data were shown as mean ± S.D. (*n* = 3).

HSD17B10 expression level has been proved to impact cell growth^22^. We established cell growth curve, CCK-8 and colony formation assays to verify that HSD17B10 knockdown HCT116 cells indeed had a slower proliferation rate (Fig. S3a–c). Re-expressing empty vector, HSD17B10 WT, 3KR and 3KQ in HSD17B10 knockdown HCT116 cells, we found that re-expression of WT HSD17B10 promoted cell growth and 3KR enhanced this effect compared to vector, while cells re-expressed with 3KQ had a slower cell growth rate compared to cells re-expressed with WT (Fig. 7a, b, c).

HSD17B10 deficiency may induce cell apoptosis and overexpression of HSD17B10 can promote cell resistance to stress^13,22^. SIRT3, the deacetylase of HSD17B10, is a stress-responsive factor as a deacetylase^23–25^. To find if HSD17B10 acetylation regulates cell resistance to stress and whether SIRT3 participates in the process, we first treated cells with H_2_O_2_, Earle’s Balanced Salt Solution (EBSS) and serum starvation (SS) which can normally induce mitochondrial dysfunction. The results showed that HSD17B10 acetylation decreased, the level of SIRT3 expression increased, and the interaction between HSD17B10 and SIRT3 became stronger under these treatments (Fig. 7d), demonstrating that SIRT3 could deacetylate HSD17B10 under stresses. Since HSD17B10 acetylation changed under stresses, we assumed that HSD17B10 acetylation might regulate cell resistance in response to stresses. We counted living cells after the treatments and verified that HSD17B10 knockdown cells lacked the resistance under stresses (Fig. S3d). With re-expression of HSD17B10 WT and 3KR, cell resistance was promoted under stresses compared to empty vector, while cells re-expressed with 3KQ were less resistant under stresses compared to WT (Fig. 7e). Taken together, HSD17B10 acetylation can affect cell growth and cell resistance in response to stresses.

## Discussion

HSD17B10 is a member of short-chain dehydrogenase family as well as an essential subunit of human mitochondrial RNase P, an enzyme responsible for mitochondrial tRNA maturation. HSD17B10 is a highly conserved gene across a large evolutionary distance, and the missense mutations cause the loss of function in dehydrogenase activity and mitochondrial tRNA maturation, resulting in HSD10MD. HSD17B10 plays an important role in mitochondrial fatty acid metabolism and mitochondrial tRNA maturation. In this study, we identified HSD17B10 as a direct target of histone deacetylase SIRT3. Hypoacetylated HSD17B10 mediated by SIRT3 increased its enzymatic activity and mitochondrial RNase P formation. This regulation resulted in increased cell growth and cell resistance in response to stresses. Our study demonstrated that acetylation of HSD17B10 regulates its functions and results in a change of cellular phenotype.

HSD17B10 is a mitochondria-located protein and our data showed that NAM, a specific inhibitor of the sirtuins, enhanced its acetylation (Fig. 3a). SIRT3, SIRT4 and SIRT5 are three sirtuins reside within the mitochondria. Overexpression of SIRT3, SIRT4 and SIRT5 can all deacetylate HSD17B10 to different degrees (Fig. 3b). However, SIRT3 had the strongest deacetylation activity to HSD17B10. Yang et al.^26^ found that there is an overlap in proteins associated with SIRT3, SIRT4 and SIRT5 and these three mitochondria sirtuins constitute the mitochondrial sirtuins network. Indeed, we also found that HSD17B10 exists in SIRT5 complex^27^. Additionally, SIRT4 could down-regulate HSD17B10 (Fig. 3b), indicating that SIRT4 may also play an important role in regulating HSD17B10 functions. These observations may be worth further investigation regarding HSD17B10 function regulation.

It has been reported that HSD17B10 can be regulated by Parkin for mitochondrial abundance in a ubiquitin-dependent manner^18^. As acetylation can stabilize the protein by inhibiting its ubiquitination^28^, it is possible that HSD17B10 acetylation could affect its ubiquitination. However, our data did not implicate that acetylation can affect HSD17B10 abundance. It seems that these two post-translational modifications do not have cross talk in regulating its functions; it could be that they are occurred at different lysine sites. Further exploration of other post-translational modifications for HSD17B10 function regulation and their disease relevance would be worthwhile.

Missense mutations identified in HSD10MD cases have been verified to impair HSD17B10 dehydrogenase activity and mitochondrial RNase P function, thus impairing mitochondrial integrity and inducing apoptosis^13,15,21^. However, these mutations do not influence the acetylation of HSD17B10 (data not shown). HSD17B10 acetylation mutants 3KR (mimic for hypoacetylation status) and 3KQ (mimic for hyperacetylation status) were proved to affect its enzymatic activity and mitochondrial RNase P formation (Fig. 5e–g). As a consequence, HSD17B10 acetylation changes cell NAD^+^/NADH ratio and the amount of mt pre-tRNAs. Also, the mitochondrial membrane potential alters with the changes of HSD17B10 acetylation, indicating the dysfunction of mitochondria. It is possible that mutations in acetylation sites of HSD17B10 could lead to HSD10MD.

HSD17B10 may contribute to the neuronal dysfunction associated with Alzheimer disease (AD) by interacting with intracellular amyloid-beta (Aβ), and binding with Aβ can inhibit HSD17B10 activity^17,29^. Our data showed that acetylation of HSD17B10 can interrupt its interaction with TRMT10C to affect the formation of mt-tRNA splicing complex (Fig. 5f, g). As protein acetylation often affects protein–protein interaction, it will be interesting to investigate if acetylation of HSD17B10 can interfere the interaction of HSD17B10 and Aβ to affect the development of AD.

Overexpression of HSD17B10 has been proved to increase cell growth and resistance to cell death^22^. As expected, HSD17B10 acetylation can regulate cell growth. SIRT3 is a stress-responsive deacetylase and we have proved that HSD17B10 acetylation plays a regulatory role in maintaining mitochondrial membrane potential. HSD17B10 acetylation levels decreased under stresses, and SIRT3 acts as the deacetylase. Deacetylated HSD17B10 protected cells from apoptosis under stresses comparing with acetylated HSD17B10 (Fig. 7d, e). Our study provides a possible mechanism for how HSD17B10 functions in cell growth and protecting cells from apoptosis. The results demonstrated that acetylation is an important regulation mechanism for HSD17B10 and may provide insight into interrupting the development of Alzheimer disease.

## Supplementary information

Supplementary information1

Supplementary information2

Supplementary information3

Supplementary information4
