# Supplementary material for: Deacetylation of HSD17B10 by SIRT3 regulates cell growth and cell resistance under oxidative and starvation stresses
Source: Cell Death Dis. 2020 Jul 23;11(7):563. doi: 10.1038/s41419-020-02763-9 (PMC7378191; doi:10.1038/s41419-020-02763-9)
Supplement: Supplementary file 1 — Supplementary information1 [file 41419_2020_2763_MOESM1_ESM.docx]

**Supplementary figure legends**

**Fig. S1 HSD17B10 acetylation affects its enzymatic activity.**

Michaelis-Menten plots and kinetic parameters for the catalysis of acetoacetyl-CoA by **(a)** deacetylated and hyper-acetylated HSD17B10, **(b)** Flag-tagged HSD17B10 WT and KR mutants and **(c)** Flag-tagged HSD17B10 WT, 3KR and 3KQ. Data were shown as mean ± S.D. (n=3).

**Fig. S2 HSD17B10 regulates the intracellular functions.**

**a** Endogenous HSD17B10 was detected in HCT116 HSD17B10 knockdown clone vector, 1o and 5a with HSD17B10 antibody. **b** The absorbance at 450nm was measure to quantify NADH and NAD_total_ in HCT116 HSD17B10 knockdown clone vector, 1o and 5a. NAD^+^/NADH= (NAD_total_-NADH)/NADH. Data were shown as mean ± S.D. (n=3). **c** The amounts of representative mt pre- and total tRNAs in HCT116 HSD17B10 knockdown clone vector, 1o and 5a were confirmed by q-PCR. Data were shown as mean ± S.D. (n=3). **d** HCT116 HSD17B10 knockdown clone vector, 1o and 5a were stained with TMRM (10nM) and Mitotracker (200nM) for 0.5h.

**Fig. S3** **HSD17B10 affects cell growth and cell resistance under stresses.**

**a** 5×10^4^ HCT116 HSD17B10 knockdown clone vector, 1o and 5a cells were seeded into 6-well plates. Cell number was counted every 24h. Data were shown as mean ± S.D. (n=3). **b** 5×10^3^ HCT116 HSD17B10 knockdown clone vector, 1o and 5a cells were seeded into 96-well plates. Cell growth was measured by CCK-8 assay after 48h. Data were shown as mean ± S.D. (n=3). **c** 5×10^3^ HCT116 HSD17B10 knockdown clone vector, 1o and 5a cells were seeded into 6cm plates. Colony formation was shown after crystal violet staining. **d** 2×10^5^ HCT116 HSD17B10 knockdown clone vector, 1o and 5a cells were seeded into 6-well plates. After 24h, cells were treated with different stresses for the indicated time, and the number of living cells was counted. Data were shown as mean ± S.D. (n=3).
